# A novel index of protein-protein interface propensity improves interface residue recognition

**DOI:** 10.1186/s12918-016-0351-7

**Published:** 2016-12-23

**Authors:** Wentao Dai, Aiping Wu, Liangxiao Ma, Yi-Xue Li, Taijiao Jiang, Yuan-Yuan Li

**Affiliations:** 1 Shanghai Center for Bioinformation Technology, 1278 Keyuan Road, Shanghai, 2012035 People’s Republic of China; 2 Suzhou Institute of Systems Medicine, Suzhou, Jiangsu 215123 China; 3 Shanghai Industrial Technology Institute, 1278 Keyuan Road, Shanghai, 201203 People’s Republic of China; 4 Shanghai Engineering Research Center of Pharmaceutical Translation, 1278 Keyuan Road, Shanghai, 201203 People’s Republic of China; 5 Center for Systems Medicine, Institute of Basic Medical Sciences, Chinese Academy of Medical Sciences and Peking Union Medical College, Beijing, 100005 China

## Abstract

**Background:**

Protein-protein interface holds important information of protein-protein interactions which play key roles in most biological processes. In the past few years, a lot of efforts have been made to improve interface residue recognition by characterizing protein-protein interfaces and extracting relevant features. However, most previous studies were carried out in a qualitative level, and there are also some inconsistencies between them.

**Results:**

In the present work, to improve interface residue recognition, we built a novel quantitative residue protein-protein interface propensity index (QIPI) and gained a comprehensive picture of protein-protein interface through analyzing protein-protein interfaces on our comprehensive protein-protein interfaces dataset (Astral2.05-40-4506). Furthermore, in order to assess the effect of QIPI in improving the protein-protein interface prediction, we developed an interface residue recognition method SPR (Single domain based Patch Recognition) based on the QIPI. The evaluation results proved that our novel QIPI is able to improve the interface residue recognition.

**Conclusions:**

Through a comprehensive quantitative analysis of protein-protein interface, we constructed a novel quantitative protein-protein interface propensity index (QIPI), which could be easily applied to improve the interface residue recognition and helpful in understanding the protein-protein interface.

**Availability:**

QIPI and SPR are available to non-commercial users at our website: http://www.scbit.org/QIPI/.

**Electronic supplementary material:**

The online version of this article (doi:10.1186/s12918-016-0351-7) contains supplementary material, which is available to authorized users.

## Background

Protein-protein interactions play crucial roles in many biological functions [1–3]. A detailed characterization of protein-protein interactions may provide crucial information about the function of protein complexes which would be helpful in medicine and drug researches [4–6]. In order to elucidate the mechanisms of protein-protein interactions, a number of biophysical techniques [7, 8] including X-ray crystallography, various spectroscopic techniques, cross-linking methods, mutation studies and so on, have been employed to investigate protein-protein interface properties. Meanwhile, a lot of efforts have been made to find the critical factors determining the specificity and affinity of protein–protein interfaces [3, 9–11].

It is indicated that protein-protein interfaces are characterized by several distinguishing properties from the rest of the surfaces in terms of geometric and chemical complementarities between interfaces, ranging from hydrophobic forces, electrostatic forces, surface planarity, interface biased residue composition to inter-residue contacts [12–15]. Knowledge of these characteristics has enabled the understanding of the interface as a whole. Various hypotheses have been proposed to delineate the interface architecture and explore the mechanisms of protein-protein interactions. The first study is O-Ring theory which concluded that the existence of a hot-spot enriched region at the center surrounded by an outer ring of non-conserved residues to occlude water [16, 17]. Later on, a series of hypotheses were developed to refine the O-Ring theory [18–20]. Another viewpoint proposes that interface should be divided into core and rim area: the former consisting largely of buried atoms and the latter formed mainly by exposed atoms [21]. However, there are some inconsistencies between these studies. Taking basic residues’ interface preference as an example, Arg and His showed positive interface propensity in some studies [14, 15] but opposite preference of these ones were also reported by other researchers [21, 22]. Moreover, qualitative results were given by most previous studies, while the interface residue recognition methods essentially need quantitative interface propensities [14, 15, 23]. There are two main reasons leading to these contradictory conclusions in previous studies: lacking a comprehensive non-redundant protein-protein interface dataset and ignoring the bias effect of solvent accessibility between interfaces and non-interface surfaces. In order to gain a comprehensive picture of protein-protein interface, we first constructed a latest comprehensive protein-protein interfaces dataset (Astral2.05-40-4506) which was extracted from the latest version of Structural Classification of Proteins — extended (SCOPe) database (v2.05) [24]. Then we reassessed the various features excluding the bias effect of solvent accessibility in a suitable manner on the dataset Astral2.05-40-4506.

In this work, we performed a novel analysis of protein-protein interface on our comprehensive protein-protein interfaces dataset (Astral2.05-40-4506). Because the interface and non-interface surfaces have different solvent accessibility, it is not well known whether their difference is due to the differences in solvent accessibility or differences in functionality (such as protein-protein interaction). The bias effect of solvent accessibility should be excluded in the protein-protein interface analysis. We analyzed the interface using non-interface surface as reference to remove the bias effect of solvent accessibility. In a convincing manner, a novel quantitative residue interface propensity index (QIPI) was constructed from our analysis and an interface residue recognition method SPR (Single domain based Patch Recognition) was developed based on the quantitative index to evaluate the interface prediction power of QIPI. The result shows that the QIPI not only characterizes protein-protein interfaces, but also helps to improve the interface residue recognition.

## Methods

### Datasets and interface definition

Protein complexes were retrieved from the latest version of Structural Classification of Proteins — extended (SCOPe) database (v2.05) [24]. A previous study demonstrates that interface properties showed consistency across different datasets, which are from the same raw protein database but with different constraints on sequence similarity and structure quality [14]. Based on the above reason, we constructed the Astral2.05-40 dataset, which is a subset of SCOPe2.05 with less than 40% identity between any two domains, for large-scale analysis of interface propensities.

A dataset of protein-protein interfaces (referred to as Astral2.05-40-4506), which consists of 4506 interfaces, was thus obtained from the Astral2.05-40 dataset.

The Astral2.05-40-4506 was used as the comprehensive interface dataset to analyze characteristics of protein-protein interfaces and develop our interface prediction method. We used the independent dataset Docking Benchmark 2.0 [25] to evaluate the power of new interface features especially the quantitative residue interface propensity index (QIPI) for interface prediction. The Docking Benchmark 2.0, which contained 84 complexes and 168 monomers, consists of 168 interfaces.

Two protein-protein interface datasets were widely used to assess interface residue recognition methods in the previous study. The first dataset consists of 25 CAPRI targets and 176 interfaces. The second dataset Enz35 set consists of 35 protein interfaces [26] and these proteins in this dataset are all enzymes. In order to compare SPR with the existing popular interface prediction method directly, we carried out the tests based on these two datasets.

For a single domain, the residue whose accessible surface area (ASA) > 1 Å^2^ is defined as surface residue. Surface residues were classified into two groups: interface and non-interface. The interface is formed by spatially neighboring residues whose ASA between single domain and complex were changed more than 1 Å^2^ per site and cross-interface contacts distance < 5 Å. The other surface residues are non-interface [14, 26, 27]. The accessible surface area (ASA) of residues was computed using NACCESS (http://www.bioinf.manchester.ac.uk/naccess/). Only surface residues were considered in the analysis and assessment. Similarly, only unbound structures were used for interface prediction.

### Relative Interface Ratio (RIR) and contact preferences

Let *f*_*i*_ be the number of interface residues of type i, and *F*_*i*_ be the number of non-interface surface residues of type i. The *frequency* of residue i in the interface and non-interface surface were calculated as *w*_*i*_ = *f*_*i*_/∑_*m*_ *f*_*m*_ and *W*_*i*_ = *F*_*i*_/∑_*m*_ *F*_*m*_ (m is the residue type), respectively. The *relative interface ratio* (RIR) of residue type i was given by (*w*_*i*_ */W*_*i*_). As the similar criteria, we analyzed the frequency and RIR of secondary structure elements in interface. In order to analyze the independent and cooperation effect of residues and secondary structures, we considered 60 classes of residues as defined by 20 residue types multiplied by 3 secondary structure states and analyzed the frequency and RIR of the 60 kinds of residues at interface.

In order to describe the ASA propensities for interface and non-interface surface residues, we got the ASA threshold *A*_*t*_ for residue type i from the Astral2.05-40-4506. The ASA threshold *A*_*t*_ was defined that ASA frequency (percentage of residues in the ASA bins) of interface residue type i was very close to the ASA frequency of non-interface surface ones in the *A*_*t*_ bin (Additional File 1: Figure S1). The *A*_*t*_ of 20 amino acids were calculated and shown in Additional file 2: Table S1. *f*_*IS(i)*_was the number of interface residue type i whose ASA < *A*_*t*_, and *f*_*IL(i)*_ was the number of interface residue type i whose ASA ≥ *A*_*t*_. As the similar definition, the *f*_*SS(i)*_ and *f*_*SL(i)*_ are generated for the non-interface surface residue type i. The *relative interface ratio* (RIR) of residue type i in ASA was given by (*f*_*IL*(*i*)_/*f*_*IS*(*i*)_)/(*f*_*SL*(*i*)_/*f*_*SS*(*i*)_).

*C*_*ij*_ was the number of interface-crossing contacts between residues of types i and j. The raw contact frequency between residues of types i and j was calculated as (*C*_*ij*_/∑_*m*,*n*_ *C*_*mn*_). Here, m and n are residue types in the interface-crossing contacts. The contact preference between residue types i and j was calculated as log_2_((*C*_*ij*_/∑_*m*,*n*_ *C*_*mn*_)/(*w*_*i*_ × *w*_*j*_)), where *w*_*i*_ and *w*_*j*_ were defined as above.

Interface size and residue number is calculated separately for each side of an interface. Domain size is also calculated for each domain. The summary of statistic result was shown in histogram and probability density function curve.

### Interface prediction

Based on characteristics of interface especially the QIPI in our analysis, a novel method SPR (Single domain based Patch Recognition) was developed as an interface predictor to assess the effect of interface features founded by us. Therefore, in SPR, we focus on (i) patches generated on the protein surface as virtual interfaces, which is described in the section of patch generation and (ii) the scoring function to evaluate the quality of a virtual interface, which is described in the section of scoring function.

Patch generation on the protein surface

In the SPR algorithm, the patch generation on the protein surface follows the four steps.

- Step I: Identification of surface residues. As in the above analysis, surface residues are defined as accessible surface area (ASA) > 1 Å^2^.
- Step II: Generation of residue side-chain distance matrix. For a protein sequence, the minimum distance between side-chain atoms of each residue pair (Cα to Cα distance in the case of glycine) was calculated as the element of residue side-chain distance matrix. If the minimum distance of a residue pair >25 Å, the corresponding element in the matrix was 25 Å.
- Step III: Construction of candidate interface patches. A random surface residue was selected as the seed residue, and neighboring surface residues whose ASA and distance to the seed residue satisfy the standard in the Table 1A were included in the candidate interface patch. All of the surface residues were sampled and a series of candidate interface patches were constructed.

**Table 1:** Patch generation thresholds

A The ASA and distance with seed residue of patch residue	
Distance(Å)	ASA(> Å^2^)
(2,5)	0
(5,7)	20
(7,9)	40
(9,11)	60
(11,13)	80
(13,15)	100
B Thresholds for patch merging	
Domain ASA(Å^2^)	Identity Ratio
(0,5000)	0.8
(5000,7500)	0.7
(7500,10000)	0.6
(10000,+ ∞)	0.5
- Step IV: Merging the candidate interface patches. For candidate interface patches in a protein, two patches were merged into a new patch when the ratio of identity residues between two patches was not less than the threshold (Table 1B). The merging process was kept iterating until there wasn’t any candidate patches could be merged.

The final predicted interface is defined as the top-ranked candidate interface patch measured by the following scoring function for interface-residue recognition.

### The scoring function for interface-residue recognition

The score E_patch_ for measuring the predicting patch as an interface is a linear combination of four terms: the interface preference potential for residues preference (E_res_), hydrophobic score (E_hydro_), residue conservation preference (E_cons_) and solvation score (E_sol_). That is given as follows:

1 $$ {E}_{Patch}={E}_{res}+{w}_1{E}_{hydro}+{w}_2{E}_{cons}+{w}_3{E}_{sol} $$

where w_i_ are to-be-determined weight factors, which are obtained by training on Astral2.05-40 dataset (see below). The *E*_*res*_ and *E*_*hydro*_ are used potentials from the AAindex database [28]. The AAindex database contains a series of numerical indices representing various physicochemical and biochemical properties of amino acids and pairs of amino acids. The calculation of *E*_*res*_, *E*_*hydro*_, *E*_*cons*_, and *E*_*sol*_ sees below.

1. Residue interface propensity score. We use a scoring function to calculate similarity between patch and interface based on the sum of residue interface propensity which is calculated from QIPI. The score for a given patch, whose residue interface propensity score *E*_*res*_ was calculated as:

2 $$ {E}_{res}={\displaystyle \sum_{i\in patch,r}\left(AS{A}_i\bullet RI{R}_r\right)/RE{F}_r} $$

where ASA_i_ is the relative accessible surface area of residue r at sequence position i which belongs to the patch; RIR_r_ and REF_r_ are the relative interface ratio and the reference ASA of residue type r, respectively. The RIR_r_ for 20 amino acid residues are obtained from QIPI. The REF_r_ is the element of JANJ780101 [29] in AAindex [28] for residue type r. The JANJ780101 index is based on average accessible surface area properties of amino acids; so it is used as the reference state in the term of residue interface propensity score.

2. Hydrophobic score. The term *E*_*hydro*_ is the hydrophobic score of the query patch, which is given below:

3 $$ {E}_{hydro}={\displaystyle \sum_{i\in patch,r}{H}_i} $$

where H_i_ is the hydrophobic score in the CASG920101 [30] matrix of AAindex for the residue type r at sequence position i. The CASG920101 matrix is based on structure-derived hydrophobic potential and used for representing hydrophobic score of amino acids in this potential.

3. Residue conservation score. Residue conservation was assessed by the self-substitution score based on the sequence profile. Sequence profiles were built by using PSI-BLAST [31] to search against non-redundant (NR) database with the BLOSUM62 [32] substitution matrix. The conservation score of the given patch was defined as:

4 $$ {E}_{cons}={\displaystyle \sum_{i\in patch,r}\left({C}_{ir}-{B}_{rr}\right)} $$

where C_ir_ is the self-substitution score in the position-specific substitution matrix produced by PSI-BLAST for the residue type r at sequence position i, and B_rr_ is the diagonal element of BLOSUM62 for residue type r.

4. Solvation energy score. The *E*_*sol*_ was adapted from the one used in Cyscore [33], which is formulated as follows:

5 $$ E={\displaystyle \sum_{i\in patch}\left(\frac{V_{i, out}}{V_{i, sphere}-{V}_{i, out}}\right)} $$

where V_i,sphere_ is defined as the sphere volume in the solvent accessible surface and V_i,out_ represents the volume out of the solvent accessible surface on residue i in the patch, respectively. The radius of the sphere is set to be 1.2 Å. The Cyscore is a new empirical scoring function for protein–ligand scoring and outperforms famous methods in the field. A novel curvature-dependent surface-area model of the solvation energy score contributes obviously to improve the prediction power of Cyscore. So we used this term in our interface residue recognition scoring function.

### Training and evaluation

Interface prediction has to satisfy two competing demands, covering as many real interface residues as possible, meanwhile predicting as few false positives as possible. These two demands are evaluated by coverage and accuracy, respectively. For all predictions of interface residues, the numbers of true and false positives are TP and FP, respectively. The number of real interface residues which isn’t identified by the predictor is false negative (FN). Then, the coverage is

6 $$ COV=TP/\left(TP+FN\right) $$

and accuracy is

7 $$ ACC=TP/\left(TP+FP\right) $$

The two criteria were used as the performance assessment in our study because a good interface recognition method could identify more real interface residues with less false positives.

The parameters used in SPR were trained on the Astral2.05-40-4506 dataset that consists of 4506 interfaces from domains with less than 40% identity to each other. Subsequently, the SPR was trained and optimized with a cost function (F) as follows:

8 $$ F=COV\ast ACC $$

The optimization goal was to maximize the cost function F value. This training process could balance the accuracy and coverage to avoid the overfitting of parameters. To evaluate the robustness of the SPR, a 10-fold cross-validation for SPR on Astral2.05-40-4506 dataset was carried out.

After training of SPR using the above process, the performance of SPR was tested on two datasets CAPRI25 and Enz35 using accuracy and coverage compared with several popular interface recognition programs [14, 23].

To gain an overall performance of SPR, we further tested it on two independent datasets, CAPRI25 and Enz35, by making comparison with several popular interface prediction programs, Meta-PPISP [34], con-PPISP [35], Promat [36], PINUP [37]. Meta-PPISP is probably one of most popular programs in this field and widely used as the reference method in the recent research [38]. Meta-PPISP is a meta-server built on scores from other method through linear regression. Con-PPISP combines PSI-Blast sequence profile and solvent accessibility in a neural network. Promate is a naïve Bayesian method consisting of properties such as secondary structure, atom distribution and sequence conservation. PINUP employs solvent accessible area, sequence conservation and side-chain energy in an empirical scoring function.

## Results

In this section, we first show the characteristics of protein interfaces in our analysis and develop a novel quantitative residue interface propensity index (QIPI). Secondly, we explore the contribution of the QIPI to improvement of interface-residue recognition. Finally, we demonstrate the performance of SPR by comparing it with several existing popular interface prediction programs.

### Characteristics of interface

Each protein surface was divided into two disjoint groups: interface and non-interface. Interface properties including residue composition, secondary structure, solvent accessibility, contact preference and interface size were analyzed using Astral2.05-40-4506.

### Residue composition and QIPI

**Fig. 1:**
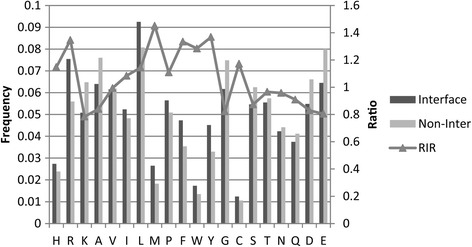
Residue composition and RIR of different amino acids. The x-axis is residue types ordered based on chemical properties (basic:H-K, hydrophobic:A-W, polar:Y-Q and acidic:D-E) and aromatic residues (P-Y) in together. In each chemical property group, residue types are ordered based on the length of residue side chain in ascending. The main y-axis is residue frequency and secondary y-axis is relative interface ratio (RIR). The frequencies of residues on interface and non-interface surface are shown in black and gray columns, respectively. The RIR is shown in triangle and line

Figure 1 compares the residue compositions of interfaces and non-interface surfaces. The comparisons show that the interfaces have more aromatic residues (Tyr, Trp, and Phe), hydrophobic residues (Met, Ile, Leu, Pro and Val), basic residues (Arg, His) and Cys than do the non-interface surfaces. In contrast with non-interface surfaces, interface preference residues also have various physical and chemical properties, but they have long side chains in average. This indicates that residues with long side chain are preferred in interfaces and disfavored for non-interface surfaces.

We calculate the *relative interface ratio* (RIR) of residues by comparing the residue composition of the interfaces with that of the non-interface surfaces. Figure 1 shows that RIR reveals that the hydrophobic residues (A-W) are more preferred at interfaces than polar residues (Y-Q) and aromatic residues (P-Y) are more frequent at interfaces. The result also shows that interfaces have high preferences for residues with long side chain. The Arg, Phe, Met, Trp and Tyr have significantly high interface propensity overall. We construct the quantitative residue interface propensity index (QIPI) from the RIR of amino acid as Table 2.

**Table 2:** Quantitative residue interface propensity index

H	R	K	A	V	I	L	M	P	F
1.147	1.346	0.784	0.841	0.994	1.084	1.144	1.451	1.109	1.334
W	Y	G	C	S	T	N	Q	D	E
1.284	1.368	0.823	1.172	0.873	0.966	0.958	0.909	0.830	0.805

### Secondary structure

**Fig. 2:**
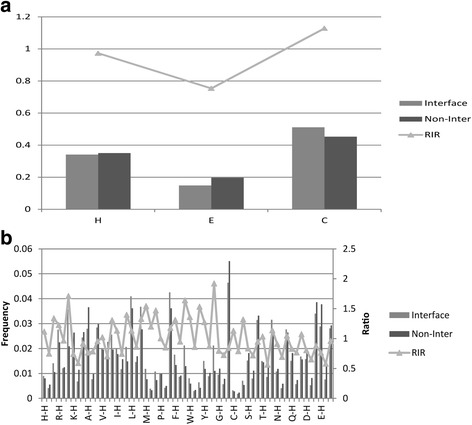
Comparison secondary structure and residue preference between interfaces and non-interface surfaces. The frequencies of secondary structure and residues on interface and non-interface surface are shown in gray and black columns, respectively. The RIR is shown in triangle and line. **a** Secondary structure composition and RIR. The x-axis is secondary structure types (H:helix, E:strand, C:coil). The y-axis is frequency and relative interface ratio value. **b** Composition and RIR of 60 classes residues. The x-axis is residue types (secondary structure combined with amino acid). The main y-axis is residue frequency and secondary y-axis is relative interface ratio (RIR)

The secondary structures are represented simply by three states: helix (H), strand (E) and coil(C). Fig. 2a compares the secondary structure compositions of interfaces and non-interface surfaces. The comparisons show that, among the three classes, strand (E) residues of interfaces have the lowest fractions and significant negative interface propensity which is measured by RIR. The opposite trend is observed for the class C (coil). No obvious preferences are observed for the helix (H). The class E and H interface preference were also reported in Yan’s previous work [14], and the preference of class C in our analysis was observed by Raih et al. [39].

Figure 2b compares the 60 residue compositions of interfaces and non-interface surfaces in order to analysis the independent and cooperation effect of residues and secondary structures. Combined with Figs. 1 and 2, we could find that the principal factor of interface propensity is the residue type. Within each residue types, trends of three secondary structure classes are almost as similar as that in Fig. 2a.

In summary, the residue composition is a crucial interface feature and the QIPI could be used in improving the interface-residue recognition.

### Solvent accessibility and contact preference

**Fig. 3:**
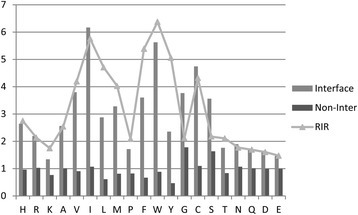
ASA propensities and RIR of residues in interface and non-inter surface. The raw ratios of residue ASA on interface and non-interface surface are shown in gray and black columns, respectively. The RIR is shown in triangle and line. The x-axis is residue types ordered based on chemical properties (basic:H-K, hydrophobic:A-W, polar:Y-Q and acidic:D-E) and aromatic residues (P-Y) in together. In each chemical property group, residue types are ordered based on the length of residue side chain in ascending. The y-axis is raw ratio and relative interface ratio value

In order to analyze solvent accessibility, ASA propensities of interface and non-interface surface residues are compared in Fig. 3. As the above definition, raw ratios of ASA for interface residues are more different than that for non-interface surface residues. The *relative interface ratio* (RIR) of residue type i in ASA was calculated by comparing ASA propensities between interface and non-interface surface residues. The RIR results show that the percentage of interface residues with larger ASA are more than that of non-interface surface ones as the above threshold *A*_*t*_. The solvent accessibility features of residues may be used in generating candidate interface patches for interface prediction.

**Fig. 4:**
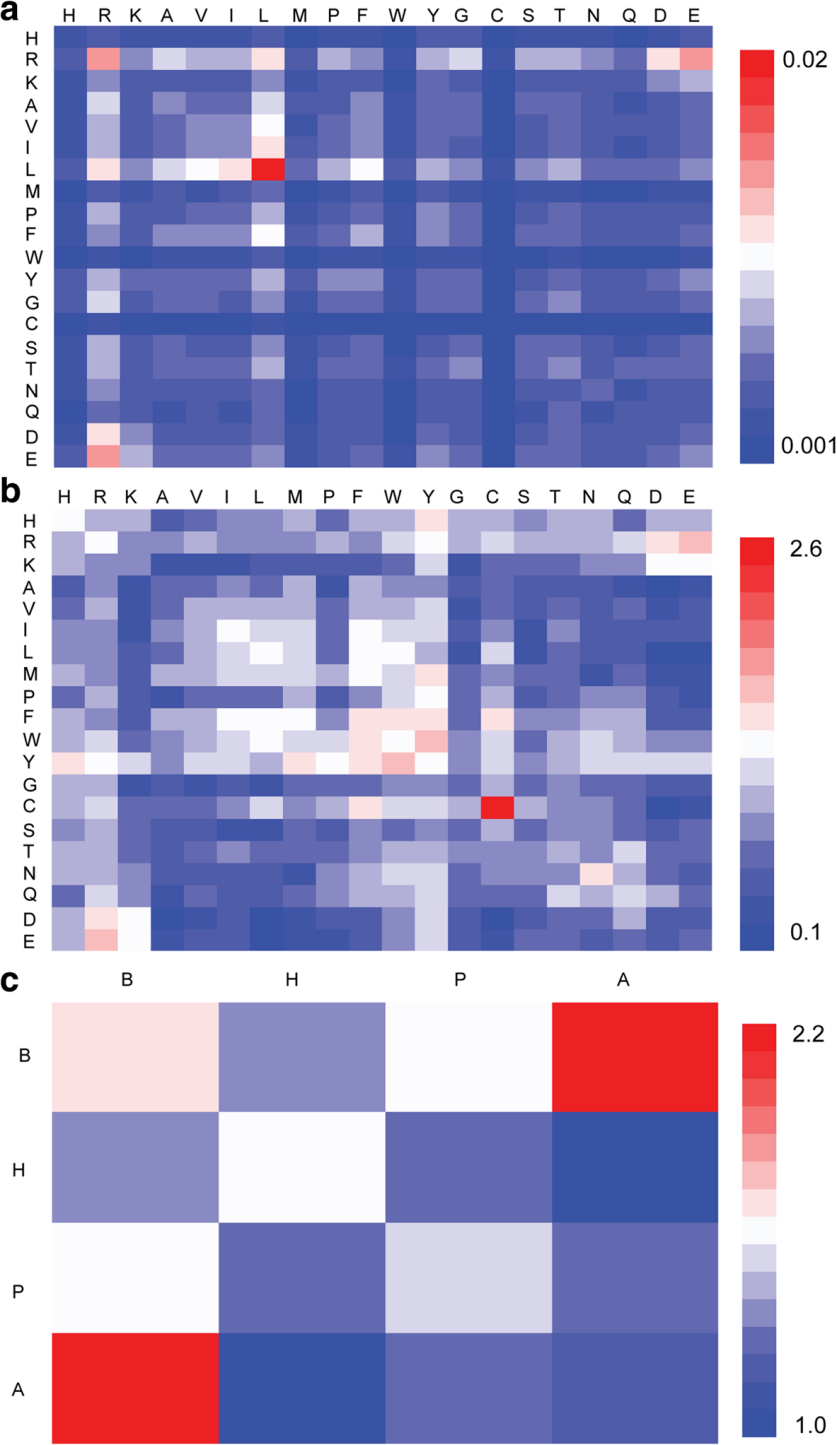
Residue contact preferences for interfaces. **a** Contact frequencies between residues of types i and j. **b** Contact preferences between residues of types i and j. **c** Contact preferences between four group residues (B:basic, H:hydrophobic, P:polar, A:acidic). In **a** and **b**, residue types ordered based on chemical properties (basic:H-K, hydrophobic:A-W, polar:Y-Q and acidic:D-E) and aromatic residues (P-Y) in together. In each chemical property group, residue types are ordered based on the length of residue side chain in ascending. These interface prefer contacts are shown in red and the opposite contacts are shown in blue

In Fig. 4, the positive contact preferences across interfaces were shown in red, negative in blue and neutral in white. Figure 4a shows the contact frequency across the interfaces given by (*C*_*ij*_/∑_*m*,*n*_ *C*_*mn*_), where *Cij* is the number of contacts formed by residues of types i and j. Figure 4b shows the contact preference given by log_2_((*C*_*ij*_/∑_*m*,*n*_ *C*_*mn*_)/(*w*_*i*_ × *w*_*j*_)), where *wi* and *wj* are frequencies of residue types i and j, respectively. In Fig. 4c, interface residues were classified into four groups: basic (B), hydrophobic (H), polar (P) and acidic(A). The contact preferences between the four group interface residues were given by the above definition and shown in Fig. 4c. Comparison of Fig. 4a and b shows that the raw contact frequency normalized by frequencies of individual residue types makes the high preferences for hydrophobic contacts (A-W), aromatic contacts (P-Y : Phe-Cys, Phe-Phe, Phe-Trp, Phe-Tyr, Trp-Tyr, Tyr-His, Tyr-Lys and Tyr-Met) and the contacts between oppositely charged residues (Arg-Asp, Arg-Glu) have been very noticeable. This observation was also supported by the Fig. 4c.

Combined with the RIR of residues and contact preferences, we may conclude that Arg, Phe, Trp and Tyr have the highest interface propensity. The reason is that RIR of these residues >1.2 (as shown in Table 2) and the number of contacts include these residues with high contact preference (more than 1.5 in pink as Fig. 4b) is at least 2. This result further supports that our QIPI grasping the interface feature.

### Interface Size

**Fig. 5:**
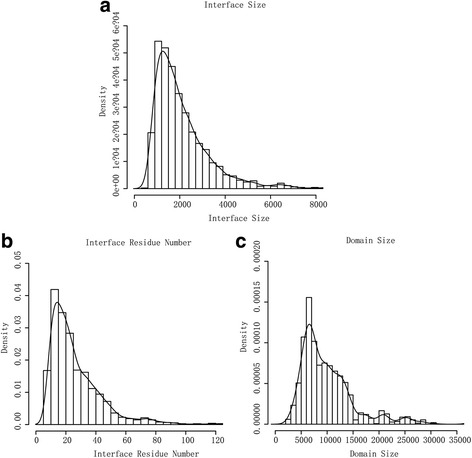
Distributions of interface size, interface residue number and domain size. **a** Interface size distribution. The x-axis is interface size (Å^2^). The y-axis is the density (fraction of interfaces). The line is the probability density function curve of interface size. **b** Interface residue number distribution. The x-axis is interface residue number. The y-axis is the density (fraction of interfaces). The line is the probability density function curve of interface residue number. **c** Domain size distribution. The x-axis is domain size (Å^2^). The y-axis is the density (fraction of domains). The line is the probability density function curve of domain size

Figure 5a shows that interface sizes span a broad range and have a gamma distribution. The average interface size is about 800 Å^2^. As shown in Fig. 5a, there are about 86% of interface sizes in the range of 0-2000 Å^2^.

In Fig. 5b, we could find that the size of interface residue number also has a gamma distribution and the average of interface residue numbers is about 20. Figure 5c shows that domain sizes also span a broad range but have a distribution that is very different from interface ones. The average domain size is about 9000 Å^2^ which is much larger than that of interface. The difference between interface and domain sizes indicates that the interface size and residue number could be used as constraints in generating candidate interface patches for prediction methods.

### The QIPI contributes to the improvement of interface residue recognition

To investigate the contribution of different interface features of SPR scoring function to the improvement of interface residue recognition, simple scoring functions with individual term and the complete scoring function were all trained on the Astral2.05-40-4506 and tested on the Docking Benchmark 2.0. The residue interface propensity which is built on the QIPI shows clearly the most effective interface prediction power (F-score = 0.089). As shown in Table 3, two terms including QIPI and hydrophobic, contributed significantly to interface residue recognition. The coverage and accuracy could be improved by QIPI (Coverage = 0.472) and hydrophobic term (Accuracy = 0.238), respectively. The performance of “QIPI + Hydrophobic” and “All-QIPI”(all features excluded QIPI) in Table 3 also suggested QIPI play an important role in the combination of features used in the interface residue recognition and its main contribution in improving the coverage. As expected, after incorporating all features, the result of complete scoring function has the best performance as F-score (0.092) which is much larger than others. At the same time, coverage and accuracy of SPR scoring function were all close to the best result.

**Table 3:** Contribution of interface features to interface residue recognition

	Coverage	Accuracy	F
QIPI	0.472	0.188	0.089
Hydrophobic	0.321	0.238	0.076
Conservation	0.266	0.191	0.051
Solvation	0.147	0.160	0.023
QIPI + Hydrophobic	0.467	0.186	0.087
All-QIPI	0.312	**0.239**	0.075
All	**0.475**	0.194	**0.092**

Note: Bold values denote the best performance in each category

To evaluate the robustness of SPR, a 10-fold cross-validation was carried out on the training set Astral2.05-40-4506. The average of coverage and accuracy were 0.506 ± 0.020 and 0.267 ± 0.019 respectively (see Additional file 2: Table S2 for details), which indicates the stable performance of SPR in the recognition of interface residue.

### Comparison of interface prediction methods

Tables 4 and 5 show the test result of five programs in CAPRI25 and Enz35 dataset respectively. The SPR achieves the highest accuracy of 0.34, the second best coverage in CAPRI25 as shown in Table 4, and the most coverage of 0.58 but the lowest accuracy in Enz35 as shown in Table 5. The result also illustrates that, on the two independent datasets, SPR which is based on the QIPI and other characteristics of interface in our study has comparable performance to the four popular interface prediction programs especially in the coverage as criterion. The performance of SPR demonstrates that characteristics of interface especially the novel quantitative residue interface propensity index (QIPI) extracted from our analysis are helpful to improve interface residue recognition.

**Table 4:** Comparisons of SPR with several popular interface prediction programs on CAPRI25 dataset

	ACC	COV
SPR	**0.34**	0.4
Cons-PPISP	0.26	0.3
Meta-PPISP	0.28	0.39
Promate	0.26	0.3
PINUP	0.25	**0.43**

Note: Bold values denote the best performance in each category

**Table 5:** Comparisons of SPR with several popular interface prediction programs on Enz35 dataset

	ACC	COV
SPR	0.36	**0.58**
Cons-PPISP	0.36	0.5
Meta-PPISP	**0.48**	0.55
Promate	0.4	0.45
PINUP	0.47	0.53

Note: Bold values denote the best performance in each category

## Discussion

In this study, through exploring the structural and physicochemical characteristics underlying various protein-protein interfaces, we have attempted to investigate various interface features and have successfully constructed a novel quantitative index of residue interface propensity. Identifying key features of protein-protein interface is a crucial step in understanding protein-protein interactions and exploring the function and evolution of protein complexes. At the same time, the quantitative interface propensity could also be used in improving the interface residue recognition, which is important for a series of computational structure biology problems such as docking and protein design. For these reasons, a number of efforts have been devoted to characterize the interface physicochemical properties and propose hypotheses such as O-Ring to depict the mechanism of protein-protein interaction. However, previous studies were limited by lacking a comprehensive non-redundant protein-protein interface dataset and ignoring relative solvent accessibility of interface residues distributions when analyzing interface features. This leads to some inconsistencies in this field. For example, Arg and His showed diverse interface preference in different previous studies, and it is difficult to improve interface residue recognition based on the qualitative knowledge from these analyses [14, 15, 23].

In order to solve the above-mentioned problems, we carried out a new quantitative analysis for exploring various features of protein-protein interface. Compared with previous studies, the main outputs of this study included: 1) a large-scale comprehensive interface dataset Astral2.05-40-4506 for analysis; 2) novel quantitative interface propensities using non-interface surface as reference to remove the bias effect of solvent accessibility; 3) a novel quantitative residue interface propensity index (QIPI) and other interface features improving interface residue recognition confirmed by the interface prediction method SPR.

Previously, lots of researches revealed that the interfaces have more hydrophobic and aromatic residues but puzzled by the observation that Arg and His also present more frequently at interface [14, 21, 22, 40]. For example, in the work of Yan et al. [14], the normalized interface propensity of residues, which is based on the accessible surface area, is highly consistent with the data based on our RIR. They concluded that the hydrophobic and aromatic residues had high interface propensity, but they were not able to explain the high interface propensities of Arg and His. According to our analysis, it is indicated that residues with long side chain (such as Arg and His) showed interface preference in a convincing manner, which solves the above puzzle. Our observation about interface preference of hydrophobic and aromatic residues is also consistent with some previous studies. For example, Ile, Val and Leu have high positive propensities for interfaces have been reported by Bahadur et al. [40] and Yan et al. [14]. In summary, we concluded that characteristics of interface residues are as follows: hydrophobic, aromatic and long side chain. These residues could form strong driving forces, such as hydrophobic interactions, which drive the formation of protein complexes and stabilize the resulting complexes.

The interface contact preference contacts in our analysis included three types of contacts: Cys–Cys, contacts between residues with opposite charges, and contacts between hydrophobic residues. The fact that Cys–Cys contacts have one of the highest preferences indicates the important role of this type of contacts in protein–protein interactions. These results are consistent with previous reports which claimed that disulfide bonds, salt bridges, and hydrophobic interactions represent the main forces in protein–protein interactions [13, 41–44]. This is also supported by the observations that at close distances, interactions between pairs of hydrophilic residues are principally important; whereas hydrophobic interactions are crucial at longer distances [13, 42, 43, 45]. Integrated with the interface preference residues and contacts, we found that that Arg, Phe, Trp and Tyr have the highest interface propensity. The residue and contact preference in interfaces observed in this analysis are consistent with the 'Double water exclusion’ [18] which is refined from the O-Ring theory [16] and roles of interface residues in the previous reports [46, 47].

We analyzed the distributions of interface size, interface number and domain size. As shown in Fig. 5, the average interface size is approximate 800 Å^2^ and about 86% of interface sizes is in the range of 0-2000 Å^2^. Our observation is consistent with the interface size distribution reported by previous researches. In these studies, Yan et al. found that the distribution of interface sizes has a peak in the range of 600-800 Å^2^ (whose average is 1227 Å^2^) [14] and Lo Conte et al. reported that the buried area for each side of the interface is about 800 Å^2^ [48]. Compared with the interface size, the domain size has a different distribution. Our research gives a generating candidate interface patches method using the interface size, interface number and domain size as constraint as Table 1.

Based on the above results, we constructed a novel quantitative residue interface propensity index (QIPI) which could be easily applied in the interface residue recognition approach. We concluded that QIPI shows clearly the effective improvement in interface residue recognition especially the coverage but its expense is losing accuracy as shown in Table 3. In order to further confirm the interface prediction power of QIPI and other interface features in our result, we developed a protein-protein interface residue recognition method SPR based on these characteristics of protein-protein interface. Through rigorous testing on independent datasets, SPR using a simple empirical scoring function shows comparable prediction power with other four popular interface prediction programs that most belong to the machine learning method especially for the coverage criterion. SPR could be applied to most protein-protein interface but its accuracy on enzyme protein interface (Enz35 dataset) is relative poor as shown in Table 5. This result demonstrates that characteristics of protein-protein interface extracted from our analysis, especially the QIPI, are effective in improving protein-protein interface residue recognition. Through analyze the all testing result (Additional file 2: Table S2 and Tables 3 and 4), we could conclude that the main contribution of QIPI is to significantly improve the coverage of interface residue recognition, while the cost is the loss of accuracy for the competition balance between coverage and accuracy.

## Conclusion

In conclusion, we constructed a novel quantitative residue interface propensity index (QIPI) through building a comprehensive non-redundant protein-protein interface dataset Astral2.05-40-4506 and quantitatively analyzing the protein-protein interface by considering the effect of relative solvent accessibility of interface residues factors distributions. The QIPI with other interface features from our analysis was helpful to explore protein-protein interfaces, and solved some inconsistent observations in previous studies such as interface propensity of Arg and His. Moreover, the QIPI successfully improved the protein-protein interface residue recognition, which was confirmed by the contribution test (Table 3), performance of SPR (Tables 4 and 5) and 10-fold cross-validation test (Additional file 2: Table S2). Therefore, the QIPI not only depicts the protein-protein interface, but also improves the protein-protein interface residue recognition. Our work provides a systematic study of protein-protein interfaces, and we believe that the quantitative index, QIPI, will contribute to the development of protein-protein interaction research.

## Additional files

Additional file 1: Figure S1. The Frequency Distribution of ASA for residues on interface and non-interface surface. (PDF 2687 kb)
Additional file 2: Table S1. The ASA threshold (Å2) for amino acids. **Table S2.** 10-Fold Cross-Validation for SPR on Astral2.05-40-4506. (DOCX 17 kb)
