# Supplementary figures and images for: A novel index of protein-protein interface propensity improves interface residue recognition
Source: BMC Syst Biol. 2016 Dec 23;10(Suppl 4):112. doi: 10.1186/s12918-016-0351-7 (PMC5259823; doi:10.1186/s12918-016-0351-7)

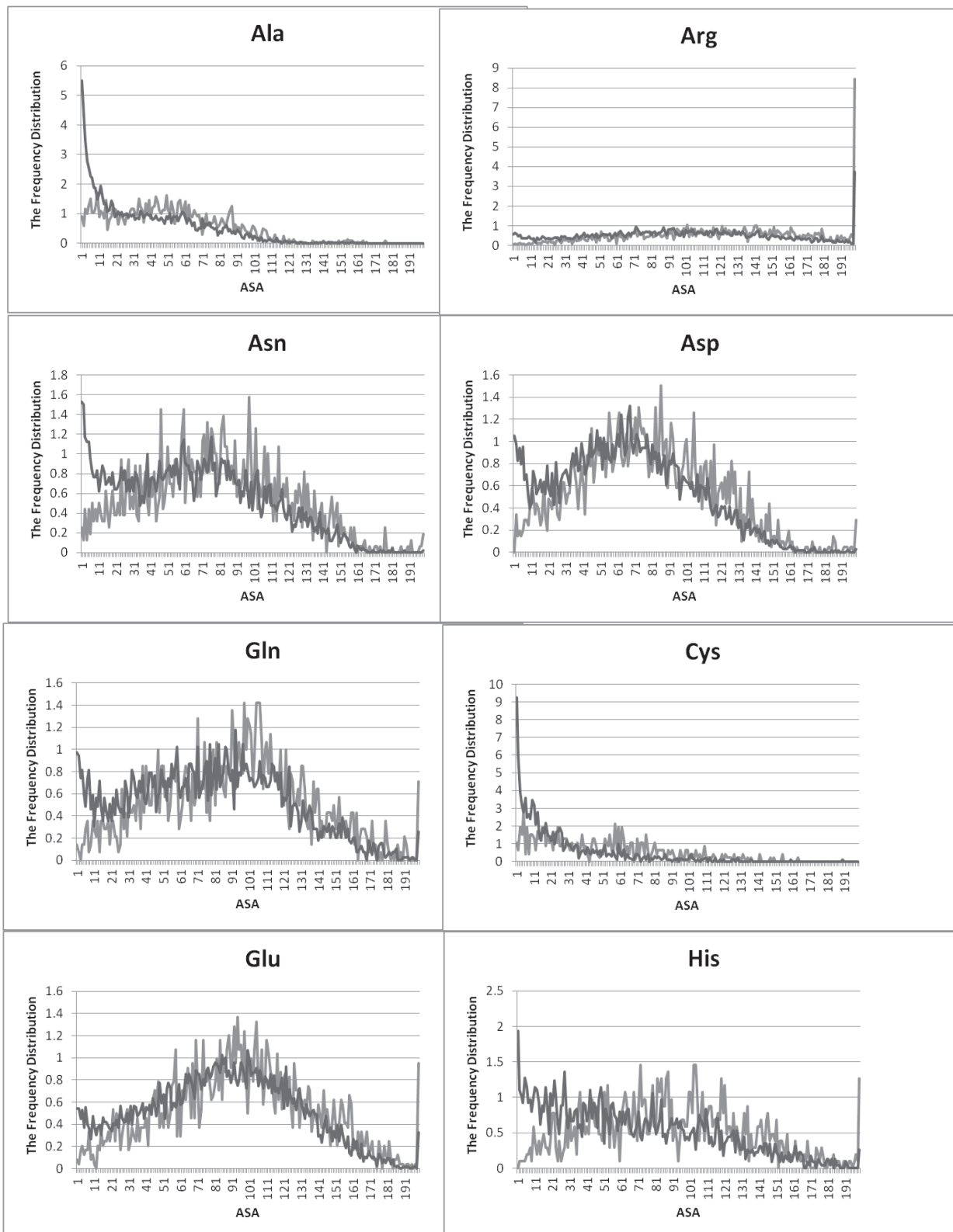

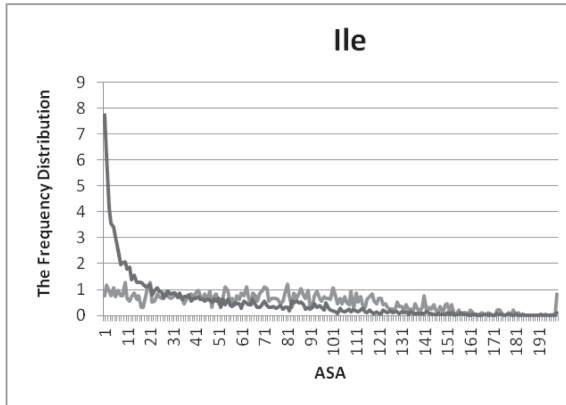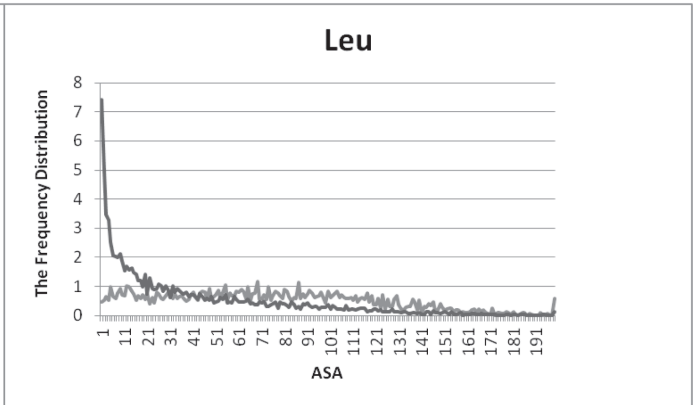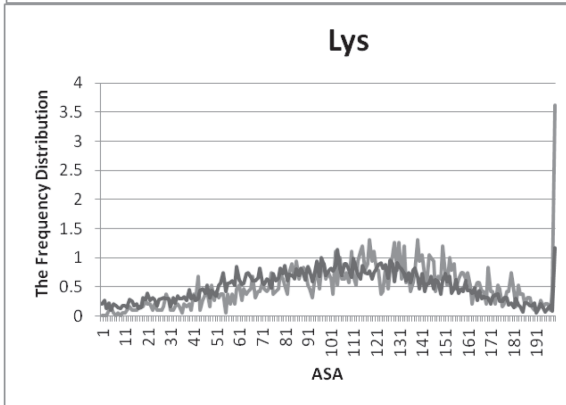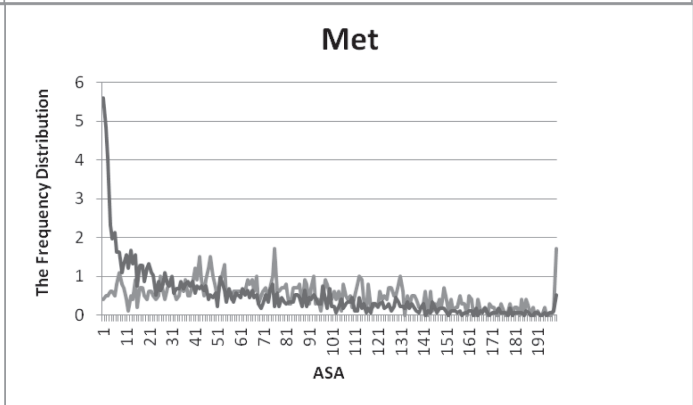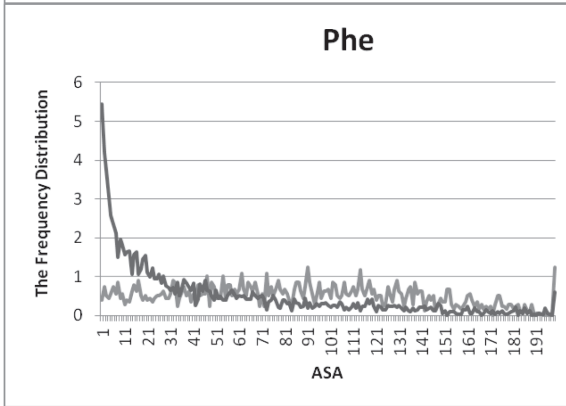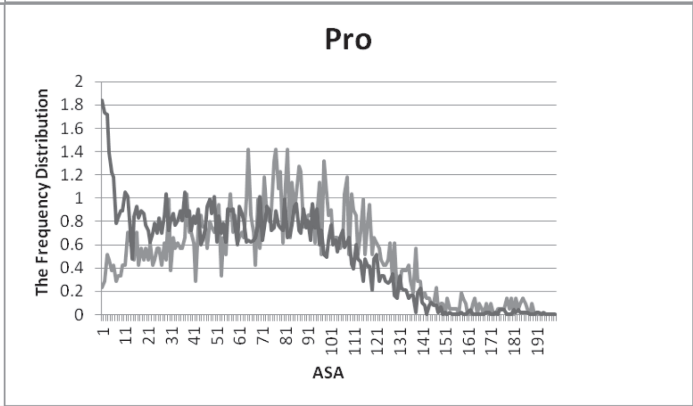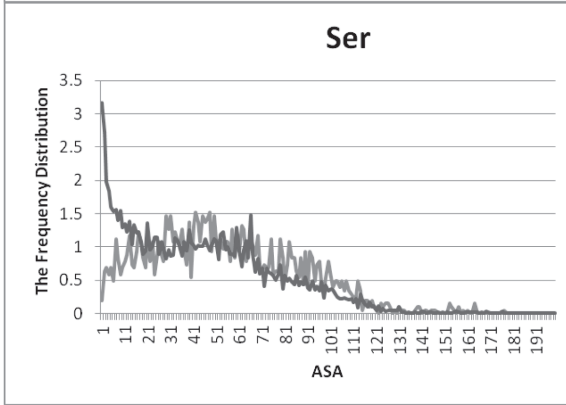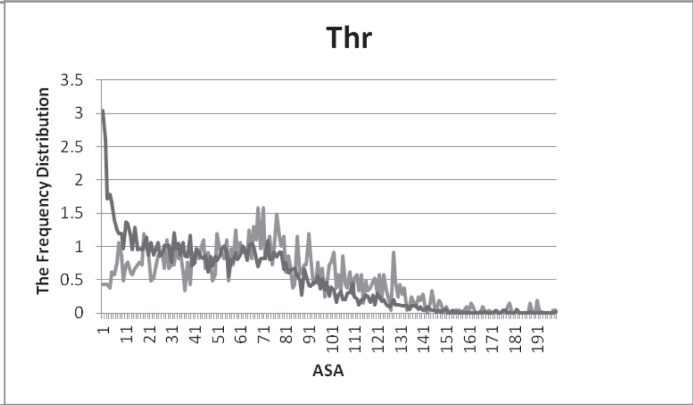

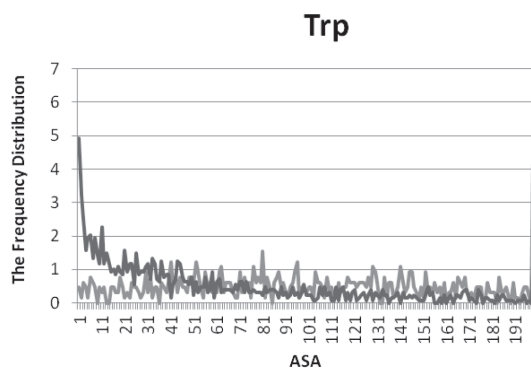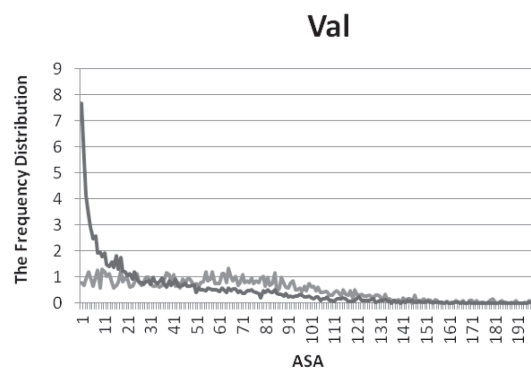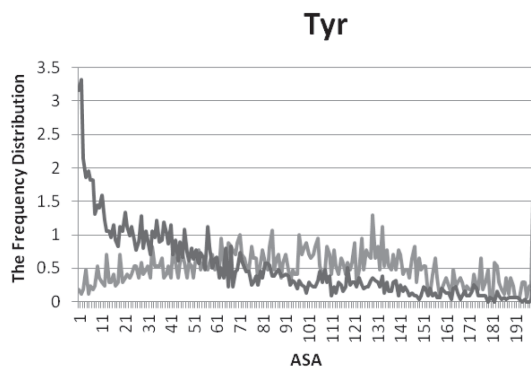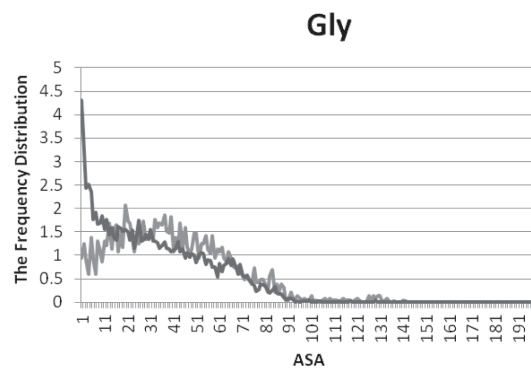

Supplement: Additional file 1: Figure S1. — The Frequency Distribution of ASA for residues on interface and non-interface surface. (PDF 2687 kb) [file 12918_2016_351_MOESM1_ESM.pdf]
