# Supplementary material for: A novel index of protein-protein interface propensity improves interface residue recognition
Source: BMC Syst Biol. 2016 Dec 23;10(Suppl 4):112. doi: 10.1186/s12918-016-0351-7 (PMC5259823; doi:10.1186/s12918-016-0351-7)
Supplement: Additional file 2: Table S1. — The ASA threshold (Å2) for amino acids. Table S2. 10-Fold Cross-Validation for SPR on Astral2.05-40-4506. (DOCX 17 kb) [file 12918_2016_351_MOESM2_ESM.docx]

Table S1 The ASA threshold (Å^2^) for amino acids

| H | R | K | A | V | I | L | M | P | F |
| --- | --- | --- | --- | --- | --- | --- | --- | --- | --- |
| 60 | 110 | 100 | 25 | 30 | 40 | 40 | 40 | 60 | 40 |
| W | Y | G | C | S | T | N | Q | D | E |
| 50 | 50 | 20 | 25 | 30 | 30 | 60 | 80 | 50 | 90 |

Table S2 10-Fold Cross-Validation for SPR on Astral2.05-40-4506

|  | Coverage | Accuracy | F |
| --- | --- | --- | --- |
| Set1 | 0.531 | 0.268 | 0.142 |
| Set2 | 0.491 | 0.280 | 0.137 |
| Set3 | 0.472 | 0.263 | 0.124 |
| Set4 | 0.503 | 0.300 | 0.151 |
| Set5 | 0.524 | 0.252 | 0.132 |
| Set6 | 0.535 | 0.262 | 0.140 |
| Set7 | 0.513 | 0.238 | 0.122 |
| Set8 | 0.488 | 0.271 | 0.132 |
| Set9 | 0.498 | 0.246 | 0.122 |
| Set10 | 0.506 | 0.286 | 0.144 |
| Mean | 0.506 | 0.267 | 0.135 |
| SD(Standard Deviation) | 0.020 | 0.019 | 0.010 |

Figure S1 The Frequency Distribution of ASA for residues on interface and non-interface surface. The x-axis is ASA bins (10 Å^2^/bin) of residues. The y-axis is ASA frequency (percentage of residues in the ASA bins) of this residue. The frequencies (percentage of residues in the ASA bins) of ASA for residues on interface and non-interface surface are shown in gray and black lines, respectively.
